# Epidemiological features of COVID-19 patients with prolonged incubation period and its implications for controlling the epidemics in China

**DOI:** 10.1186/s12889-021-12337-9

**Published:** 2021-12-09

**Authors:** Zhi-Jie Zhang, Tian-Le Che, Tao Wang, Han Zhao, Jie Hong, Qing Su, Hai-Yang Zhang, Shi-Xia Zhou, Ai-Ying Teng, Yuan-Yuan Zhang, Yang Yang, Li-Qun Fang, Wei Liu

**Affiliations:** 1 Department of Epidemiology and Health Statistics, School of Public Health, Fudan University, Shanghai, P. R. China; 2 Key Laboratory of Public Health Safety, Ministry of Education, Shanghai, P. R. China; 3 State Key Laboratory of Pathogen and Biosecurity, Beijing Institute of Microbiology and Epidemiology, Beijing, P. R. China; 4 School of Mathematical Sciences, Beijing Normal University, Beijing, P. R. China; 5 Department of Biostatistics, College of Public Health and Health Professions, and Emerging Pathogens Institute, University of Florida, Gainesville, FL USA; 6 Department of Laboratorial Science and Technology, School of Public Health, Peking University, Beijing, P. R. China

**Keywords:** COVID-19, Prolonged incubation period, Transmissibility, Clinical severity

## Abstract

**Background:**

COVID-19 patients with long incubation period were reported in clinical practice and tracing of close contacts, but their epidemiological or clinical features remained vague.

**Methods:**

We analyzed 11,425 COVID-19 cases reported between January–August, 2020 in China. The accelerated failure time model, Logistic and modified Poisson regression models were used to investigate the determinants of prolonged incubation period, as well as their association with clinical severity and transmissibility, respectively.

**Result:**

Among local cases, 268 (10.2%) had a prolonged incubation period of > 14 days, which was more frequently seen among elderly patients, those residing in South China, with disease onset after Level I response measures administration, or being exposed in public places. Patients with prolonged incubation period had lower risk of severe illness (OR_adjusted_ = 0.386, 95% CI: 0.203–0.677). A reduced transmissibility was observed for the primary patients with prolonged incubation period (50.4, 95% CI: 32.3–78.6%) than those with an incubation period of ≤14 days.

**Conclusions:**

The study provides evidence supporting a prolonged incubation period that exceeded 2 weeks in over 10% for COVID-19. Longer monitoring periods than 14 days for quarantine or persons potentially exposed to SARS-CoV-2 should be justified in extreme cases, especially for those elderly.

**Supplementary Information:**

The online version contains supplementary material available at 10.1186/s12889-021-12337-9.

## Background

The novel coronavirus disease (COVID-19), caused by severe acute respiratory syndrome coronavirus 2 (SARS-CoV-2), has spread rapidly across the world and caused a global pandemic [1]. By 1 November 2021, 246,594,191 confirmed cases and 4,998,784 deaths were reported in 223 countries, areas, or territories globally [1]. It is difficult to predict how long the coronavirus pandemic will last, due to the unequal distribution of vaccines and the changing effect of the new crown vaccines on the constantly emerging variant strains [2–5].

Non-pharmaceutical approaches including case isolation, contact tracing, and quarantine and social distancing are still and will be the main interventions for the control of this pandemic [6–8]. An accurate estimation of the length of the incubation period is crucial for highly efficient non-pharmaceutical interventions.

So far, the estimation of the median incubation period and the corresponding 95% confidence interval has been inconsistent, largely due to the heterogeneity of populations and epidemic phases that had been studied [9, 10]. Although most cases were reported with a median or mean incubation between 2 and 12 days, studies showing prolonged incubation period over 2 weeks, with an extreme incubation period of 38 days ever reported [9–13]. However, almost all previous researches were based on limited sample sizes, mostly from case reports or single case cluster that were recorded at early epidemic phase. No studies have ever focused on case series with prolonged incubation period > 14 days, to investigate their epidemiological features or to explore the association with clinical severity and transmissibility. This had raised a concern that beyond the required quarantine duration, which kind of patients were likely to transmit the disease. These need to be addressed in developing prevention and control strategies for containing the disease spread.

Here we extensively reviewed the available data of patients with known dates of exposure and symptom onset in the Chinese mainland to explore the features of patients with prolonged incubation period. The determinants for longer incubation period and the resultant impacts on the clinical severity and transmissibility of COVID-19 were evaluated for the first time as well.

## Methods

### Data sources and data extraction

Data on individual COVID-19 cases and the source transmission clusters were obtained from publicly available data, mainly from the websites of provincial and municipal health commissions in China and the Chinese Center for Disease Control and Prevention (China CDC), or through internet searches using Chinese keywords (“coronavirus” OR “pneumonia”) and (province and city names). For each identified COVID-19 case who had clear epidemiological survey information, basic demographic characteristics (age, sex, type of residence, living city), starting and ending dates of probable exposure, date of symptom onset (fever, respiratory symptoms, myalgia, etc.), date of diagnosis, date of discharge, infection route (case contact in public place or in workplace, traveling to Hubei Province, and/or household contact) were extracted as necessary information. The related epidemiological cluster were determined and likewise had epidemiological data extracted, if available. Two researchers independently reviewed the information of each case and entered the data into a standardized reporting sheet to establish a database. Discrepancies were resolved by discussion between the two researchers and facilitated by a third senior researcher to reach a consensus [14, 15].

Individual data on occupation, underlying diseases, and clinical severity were additionally collected from epidemiological investigation reports of COVID-19 cases provided by China CDC. These data were matched to the publicly available dataset by city, age, gender, reported date, and other overlapped variables. The pooled de-identified data were used for the subsequent analysis. Suspected cases and asymptomatic cases were excluded from the current study. Cases reported in Hubei Province and imported cases from abroad were also excluded due to a lack of detailed exposure information.

### Definitions of key variables

1. Incubation period: for each case *i*, let ${T}_i^E$ and ${T}_i^S$ be the exposure (infection) and symptom onset dates, respectively. The incubation period is then ${V}_i^{Inc}={T}_i^S-{T}_i^E$. The exact exposure date is usually not directly observed but rather bounded by an interval, i.e., ${L}_i\le {T}_i^E\le {U}_i$, and the incubation interval is thus bounded by ${T}_i^S-{U}_i\le {V}_i^{Inc}\le {T}_i^S-{L}_i$. We considered the earliest onset of clinical symptoms as the date of symptom onset. The date of exposure usually had the following two situations. First, if a patient had a history of travel to Hubei Province before symptom onset, the starting and ending dates of exposure (*L*_*i*_ and *U*_*i*_) were set as the dates of arriving at and departing from Hubei province, respectively. Second, if a patient was exposed to (a) a confirmed COVID-19 patient, (b) a person who resided in or had traveled to Hubei Province, or (c) a person with known contact with a confirmed COVID-19 case, the starting and ending dates of exposure (*L*_*i*_ and *U*_*i*_) were set as the initial and the last contact dates, respectively.
2. Cluster: the case clusters in our crowdsourced data were obtained by contact-tracing. All cases that were determined to be in close contact with each other were defined as a case cluster.
3. Primary and secondary cases: we use the earliest symptom onset date in each case cluster as baseline and call it day 0. A local case with symptom onset on days 0 or 1 or an imported case with symptom onset on days 0–3 is considered as a primary case; otherwise, the patient is considered a secondary case. All cases that did not belong to any cluster, were treated as primary cases.
4. Transmissibility: the number of secondary cases infected by the primary case within a cluster was a measure of the transmissibility of the primary case. If there were multiple primary cases within a cluster, they were treated as having jointly infected the secondary cases in the cluster.
5. Type of residence: Urban area or Rural area as the permanent residence.
6. Epidemic phase: Cases were assigned to a phase “Before Level I response was employed” or “After Level I response was employed” based on their onset date, with different Level I response date across different provinces.
7. Geographical Location: Northern, Southern, and Central China are defined according to the latitude of the city where the case is reported. Herein Northern China referred to north of 35°N latitude, Central China referred to between 30°N and 35°N and Southern China referred to south of 30°N.

### Estimation of incubation period

For local cases and imported cases for whom both the left and right intervals of the incubation period are complete, we respectively fitted 4 commonly used distributions of incubation period (Weibull, Gamma, Loglogistic, and Lognormal) using the package ‘fitdistrplus’ of the statistical software R. In addition, the cases were stratified according to age, duration from onset to discharge, and infection route, and estimated for the incubation periods in a disaggregated way. The optimal fitted distribution for incubation period was determined by AIC (Akaike’s Information Criterion) and was used to calculate the median of incubation period and 95% confidence interval. Based on the optimal distribution, we estimated the conditional probability that the incubation period of each case was greater than 14 days under the condition of their upper and lower intervals of the incubation period, *P*(*t* > 14| *t* > *t*_*lower*_, *t* < *t*_*upper*_). Use this probability value to randomly classify each case (includes interval-censored data and right-censored data) into a prolonged incubation period group (>14 days) or a normal incubation period group (≤14 days). We repeated this process 10,000 times, and for those with classification into prolonged incubation period group more than 5000 times, the case was grouped as with prolonged incubation period group, otherwise, the case was defined as a normal incubation period group.

### Statistical analysis

The baseline characteristics, epidemiological information, and clinical phenotype were compared between local COVID-19 cases with an incubation period of ≤14 and > 14 days. Pearson’s Chi-square test was used for categorical variables and Fisher’s exact test was used when more than 20% of cells of “R×C contingency table” have expected frequencies < 5. Wilcoxon sum-rank test was used to compare continuous variables between the two groups of patients. The changing patterns of the incubation period were profiled over four epidemic periods, by different case characteristics.

An accelerated failure time model (AFT) assuming a Gamma distribution for the incubation period was applied to evaluate the impact of patients’ characteristics on the length of incubation period. The AFT model was implemented using the “survreg” function in the R package “survival” [16]. In the “survreg” function, the parameters (baseline shape and scale parameters and covariate coefficients) were estimated via the maximum likelihood approach. This model allowed us to analyze the associations between interval-censored response variables and explanatory variables.

A Logistic regression model was used to evaluate the association between incubation period and clinical severity, with sex, age, geographical location, occupation, type of residence, underlying diseases included as covariates. Attribute value frequency (AVF) and Z-score were used to filter out outliers and be evaluated for their influence on the model [17–19].

To evaluate the impact of primary case’s characteristics on the transmissibility of COVID-19, we used the epidemiological cluster as research unit to fit the modified Poisson regression model. The number of secondary cases in a cluster was used as the dependent variable, and the characteristics of the primary cases, mainly comprised of incubation period (normal or prolonged incubation), sex, age, geographical location, type of residence, underlying diseases, epidemic phase, and clinical severity, were used as explanatory variables. If the cases did not report epidemiological association with any other confirmed cases, then transmissibility of “0” was assigned. To reduce the bias caused by incomplete epidemiological surveys information, we determined transmissibility of “0” only for cases that were reported from cities with high-quality epidemiological surveys (herein referred to the cities with over 40% of the total cases defined for their association with other confirmed cases).

Since there could be more than one primary case in a cluster, we modified the ordinary Poisson regression model to represent those multiple primary cases jointly infected secondary cases in a cluster and constructed a new logarithmic likelihood function as follows:

$$\mathit{\ln}L\left(\beta \right)=\sum_{k=1}^n\left[{y}_k\mathit{\ln}\left(\sum_{t=1}^m\mathit{\exp}\left({X_{kt}}^T\beta \right)\right)-\sum_{t=1}^m\mathit{\exp}\left({X_{kt}}^T\beta \right)-\mathit{\ln}\left({y}_k!\right)\right]$$

Where *n* represents the number of clusters, *m* represents the number of primary cases in a cluster, *X*^*T*^ is the characteristics of primary cases, *y*_*k*_ represent the number of secondary cases in a cluster. The maximum likelihood estimation was used to estimate the regression coefficients of this model.

To assess multicollinearity among the model predictors, variance inflation factor (VIF) of each variable was calculated and all VIFs in our models were lower than 2, indicating a very low multicollinearity of them (Supplementary Table 1). All the analyses were performed using R software (version 3.6.3, R Foundation for Statistical Computing, Vienna, Austria).

## Results

### Demographic characteristics of the included patients

**Fig. 1:**
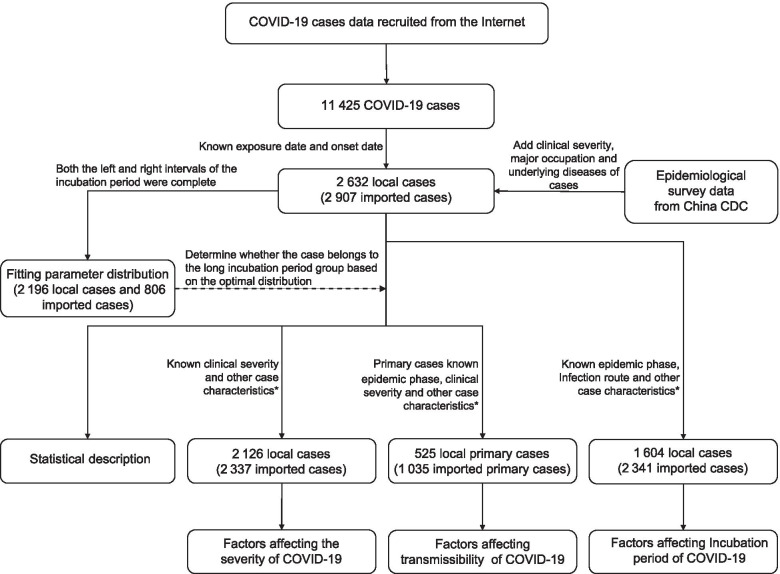
Flowchart of COVID-19 patients screened for data analyses in this study. ^*^Other case characteristics referred to sex, age, occupation, geographical location, type of residence, and underlying disease

Altogether 11,425 confirmed COVID-19 cases were included in our dataset, accounting for 81% of total cases which were reported from all 30 provinces outside Hubei Province in the Chinese mainland during the period from 20 January 2020 to 14 August 2020 (Supplementary Fig. 1). Due to the wide disparity of incubation period between imported cases from Hubei Province and local cases, we made separate evaluations and modeling for these two groups of patients. After excluding cases with missing, vague, or conflicting exposure history or unknown date of symptom onset (5886 cases), there were 2632 local cases, and 2907 imported cases were included in the final analysis. Depending on the availability of critical variables for separate analysis, 2196 local cases and 806 imported cases were used for estimation of the incubation period, 1604 local cases and 2341 imported cases were applied to estimate the impacting factors of incubation period, 2126 local cases and 2337 imported cases were analyzed for the association between incubation period and clinical severity, and 525 local primary cases (467 clusters, 412 secondary cases) and 1035 imported primary cases (984 clusters, 477 secondary cases) were analyzed for the association between incubation period and transmissibility (Fig. 1).

### Estimation of incubation period and the impacting factors

**Fig. 2:**
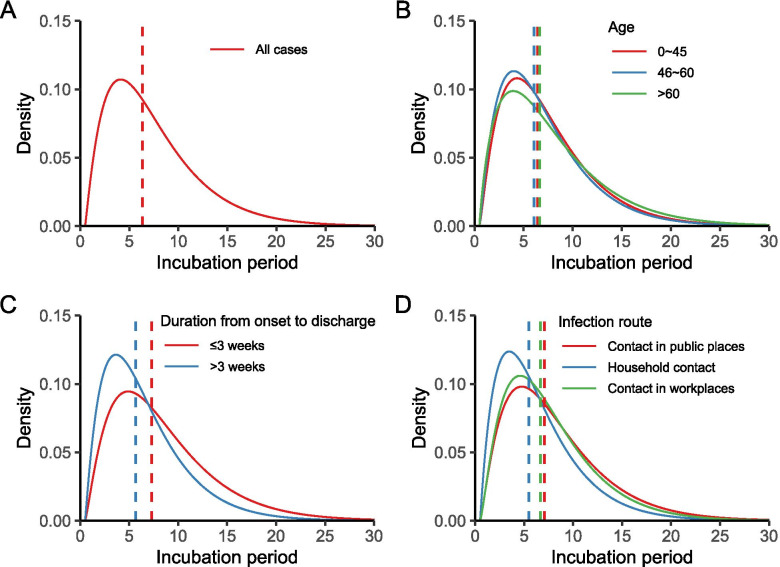
Estimated incubation period of local COVID-19 cases based on Gamma distribution. **A** The incubation period distribution of all cases. **B** The incubation period distribution of different age groups. **C** The incubation period distribution of different time groups from onset to discharge. **D** The incubation period distribution of different infection routes. Vertical lines indicate median of the Gamma distribution

Based on data from 2196 local cases, the Gamma distribution model yielded the best-fitted estimation for incubation period (median 6.4, 95% CI: 6.1–6.6 days) (Fig. 2A, Supplementary Figs. 2, 3 and 4). The elderly > 60 years seemed to have a longer incubation period than those 46–60 years (*P* = 0.003) (Fig. 2B). Patients with a longer duration from onset to discharge (> 3 weeks) had a shorter incubation period than those with the duration of ≤3 weeks (5.6 vs. 7.3 days of the median incubation period, *P* < 0.001) (Fig. 2C). Patients had a shorter incubation period for those infected by a household member than those infected by other people (median of 5.5 days vs. 7.1 days in a public place and 6.7 days in a working place, *P* < 0.001) (Fig. 2D). In the analysis of imported cases, a longer incubation period was observed from imported cases than from local cases (6.9 days vs. 6.4 days, *P* < 0.001), and all the above-mentioned inter-group differences were similarly observed (Supplementary Figs. 5, 6, 7 and 8).

For the analysis among local cases, 268 cases (10.2%) had a prolonged incubation period of > 14 days (Table 1). When compared with those with a normal incubation period within 14 days, they were more likely to be older than 60 years (*P* = 0.002), residing in Southern China (*P* = 0.002), to be secondary cases (*P* = 0.009), with non-severe disease (*P* = 0.015), to have a shorter duration from symptom onset to discharge (*P* < 0.001), to develop the disease after Level I response measures administration (*P* = 0.001), infected by being exposed in public places (*P* < 0.001). In terms of gender, occupation, type of residence, disease outcome, or underlying disease, no sufficiently credible differences between groups were observed. Among the imported cases from Hubei, differential associations were inferred. A longer incubation was associated with a higher proportion of residing in rural areas (*P* = 0.030) and with occupation types of service worker or unemployment (*P* = 0.046). In contrast, no difference between Northern and Southern China was observable (*P* = 0.157) (Supplementary Table 2).

**Table 1:** Demographic, clinical, and epidemiologic characteristics compared between local COVID-19 cases with an incubation period of ≤14 and > 14 days, in the mainland of China

Characteristics	Number of cases (%)		***P***-value^**#**^
	≤14 days	>14 days	
**Sex**			0.895
Male	1167 (49.37%)	134 (50.00%)	
Female	1197 (50.63%)	134 (50.00%)	
**Age, year**			0.002
0–45	1125 (47.59%)	119 (44.40%)	
46–60	772 (32.66%)	72 (26.87%)	
> 60	467 (19.75%)	77 (28.73%)	
**Occupation**^b^			0.840
Manual worker^a^	154 (8.11%)	21 (8.97%)	
Farmer	380 (20.02%)	47 (20.09%)	
Office worker	350 (18.44%)	48 (20.51%)	
Service worker	322 (16.97%)	34 (14.53%)	
Unemployed	692 (36.46%)	84 (35.90%)	
**Geographical location**			0.002
Northern	789 (33.38%)	70 (26.12%)	
Central	968 (40.95%)	104 (38.81%)	
Southern	607 (25.68%)	94 (35.07%)	
**Type of residence**			0.107
Urban area	1534 (64.89%)	160 (59.70%)	
Rural area	830 (35.11%)	108 (40.30%)	
**Case type**			0.009
Primary cases	1306 (55.25%)	125 (46.64%)	
Secondary cases	1058 (44.75%)	143 (53.36%)	
**Clinical severity**^b^			0.015
Non-severe	1819 (89.43%)	229 (94.63%)	
Severe	215 (10.57%)	13 (5.37%)	
**Outcome**^b^			1.000
Discharge	2013 (98.72%)	240 (98.77%)	
Death	26 (1.28%)	3 (1.23%)	
**Duration from onset to discharge**^b^, median days (IQR)	21.00 (16.00, 27.00)	19.00 (15.00, 23.00)	< 0.001
**Epidemic phases**			0.001
Before Level I response	240 (10.15%)	10 (3.73%)	
After Level I response	2124 (89.85%)	258 (96.27%)	
**Underlying disease**^b^			0.837
None	1449 (72.81%)	175 (73.53%)	
Cardia-cerebrovascular disease	276 (13.87%)	38 (15.97%)	
Diabetes	91 (4.57%)	11 (4.62%)	
Other disease	284 (14.27%)	31 (13.03%)	
**Infection route**^b^			< 0.001
Contact in public places	585 (32.02%)	96 (55.17%)	
Household contact	1060 (58.02%)	65 (37.36%)	
Contact in workplaces	182 (9.96%)	13 (7.47%)	

^#^For category variables, the Chi-square test is the preferred method, however, the Fisher’s exact test was used when more than 20% of cells of “R × C table” contingency table have expected frequencies < 5. For continuous variables, the Wilcoxon sum-rank test was used

^a^Manual worker contains construction worker, factory worker, cleaner, etc.

^b^Represents that the variable contains missing values

The multivariate accelerated failure time model that was applied on local cases further disclosed that the elderly > 60 years of age, patients with disease onset after Level I response measures administration, and those were infected via exposure in public places were associated with a longer incubation period compared those exposures through household contact (*P* < 0.001), while geographic location, type of residence, occupation, and underlying diseases seem unlikely to be related to the incubation period in local cases (Table 2). By contrast, the model applied on imported cases from Hubei, disclosed different degrees of association between incubation period and occupation, geographical location, and type of residence (Supplementary Table 3).

**Table 2:** Factors associated with incubation period among 1604 cases using accelerated failure time model

Factors	N	Univariate analysis		Multivariate analysis^**#**^	
		EXP(β) (95%CI)	***P***-value	EXP(β) (95%CI)	***P***-value
**Sex**					
Female	809	1	–		
Male	795	1.030 (0.961, 1.104)	0.406		
**Age, year**					
> 60	494	1	–	1	–
0–45	367	0.960 (0.879, 1.049)	0.366	0.945 (0.868, 1.030)	0.200
46–60	743	0.903 (0.821, 0.993)	0.035	0.898 (0.819, 0.986)	0.024
**Occupation**					
Manual worker^a^	128	1	–		
Farmer	312	0.920 (0.795, 1.065)	0.266		
Office worker	288	0.973 (0.841, 1.126)	0.711		
Public service worker	249	0.945 (0.812, 1.099)	0.464		
Unemployed	627	0.980 (0.857, 1.120)	0.767		
**Geographical Location**					
Northern	513	1	–		
Central	691	0.961 (0.886, 1.041)	0.329		
Southern	400	1.025 (0.934, 1.125)	0.608		
**Type of residence**					
Urban area	1005	1	–		
Rural area	599	1.023 (0.952, 1.100)	0.529		
**Underlying disease**					
None	1149	1	–		
Cardio-cerebrovascular disease	242	0.997 (0.901, 1.103)	0.951		
Diabetes	76	0.947 (0.802, 1.119)	0.521		
Other diseases	233	0.972 (0.880, 1.072)	0.567		
**Epidemic phase**					
Before Level I response	147	1	–	1	–
After Level I response	1457	1.811 (1.618, 2.027)	< 0.001	1.825 (1.631, 2.042)	< 0.001
**Infection route**					
Contact in public places	529	1	–	1	–
Household contact	959	0.827 (0.769, 0.890)	< 0.001	0.814 (0.758, 0.874)	< 0.001
Contact in workplaces	116	0.950 (0.828, 1.089)	0.461	0.954 (0.836, 1.089)	0.485

^#^Factors with *P*-value greater than 0.1 in univariate analysis were excluded from the multivariate analysis

^a^Manual worker contains construction worker, factory worker, cleaner, etc.

**Fig. 3:**
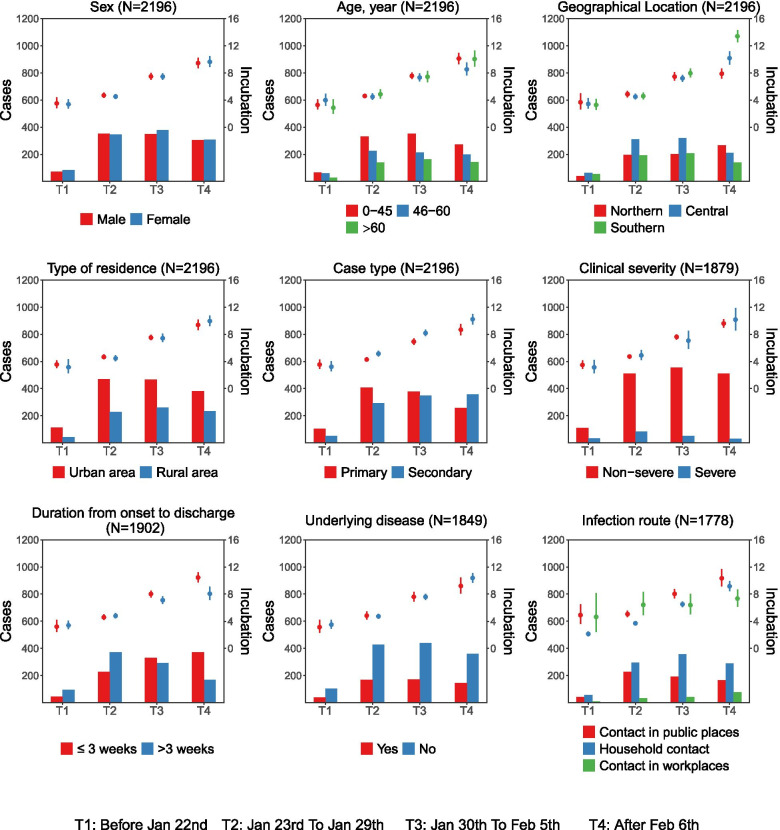
The incubation period in different populations profiled along epidemic period**.** The proportion of subgroups of patients corresponded to the left axis. The incubation period estimated from subgroups of patients corresponded to the right axis

In local cases, the median incubation period varied along the epidemic period and increased in a linear pattern from 4 days for patients being sick before 22 January 2020, to over 10 days after 6 February 2020. This temporal pattern was consistently displayed when further disseminated by age, sex, type of residence, geographical location, case type, clinical severity, duration from onset to discharge, underlying disease, and infection route (Fig. 3). For imported cases from Hubei, the incubation period likewise increased along the epidemic periods (Supplementary Fig. 9).

### Association between incubation period and disease severity

The logistic regression model was applied to evaluate the impact from incubation period on severe COVID-19 (Table 3). It’s disclosed that non-severe illness was significantly related to prolonged incubation period > 14 days than those with incubation period ≤14 days (adjusted OR = 0.386, 95% CI: 0.203–0.677), after adjusting for other factors. On the other hand, a higher risk of severe illness was related to patients > 60 years old (*P* < 0.001), residing in Northern China, with underlying diseases of diabetes. The separate analysis on the imported cases demonstrated highly similar results as that of local cases (Supplementary Table 4). The sensitivity analysis by removing outliers was shown in Supplementary Table 5, and the results show that outliers had a very weak influence on the model.

**Table 3:** Factors affecting the severity of COVID-19 among 2126 local cases using Logistic regression model

Factors	N	Univariate analysis		Multivariate analysis^**#**^	
		EXP(β) (95%CI)	***P***-value	EXP(β) (95%CI)	***P***-value
**Incubation**					
Normal	1893	1	–	1	–
Prolonged	233	0.461 (0.247, 0.790)	0.009	0.386 (0.203, 0.677)	0.002
**Sex**					
Female	1071	1	–		
Male	1055	1.058 (0.804, 1.393)	0.689		
**Age, year**					
0–45	992	1	–	1	–
46–60	670	1.615 (1.098, 2.380)	0.015	1.380 (0.927, 2.054)	0.112
> 60	464	5.857 (4.169, 8.324)	< 0.001	4.482 (3.074, 6.584)	< 0.001
**Occupation**					
Manual worker^a^	174	1	–	No significant and excluded	
Farmer	426	4.852 (2.338, 11.802)	< 0.001		
Office worker	398	1.805 (0.816, 4.566)	0.172		
Public service worker	354	2.453 (1.125, 6.149)	0.036		
Unemployed	774	3.060 (1.492, 7.389)	0.005		
**Geographical Location**					
Northern	617	1	–	1	–
Central	946	0.630 (0.463, 0.859)	0.003	0.644 (0.465, 0.894)	0.008
Southern	563	0.490 (0.333, 0.712)	< 0.001	0.561 (0.374, 0.832)	0.005
**Type of residence**					
Urban area	1291	1	–		
Rural area	835	1.212 (0.916, 1.598)	0.175		
**Underlying disease**					
None	1542	1	–	1	–
Cardia-cerebrovascular disease	305	2.562 (1.819, 3.574)	< 0.001	1.511 (1.041, 2.175)	0.028
Diabetes	101	2.457 (1.492, 3.967)	< 0.001	2.155 (1.288, 3.538)	0.003
Other disease	300	2.060 (1.457, 2.877)	< 0.001	1.605 (1.114, 2.283)	0.010

^#^Factors with *P*-value greater than 0.1 in univariate analysis were excluded in the multivariate analysis

^a^Manual worker contains construction worker, factory worker, cleaner, etc.

### Association between incubation period and transmissibility

Based on data from 467 clusters involving 525 primary cases, lower transmissibility was observed among the primary patients with prolonged incubation period, with a 50.4% (95% CI: 32.3–78.6%, *P* = 0.002) reduction from that among patients with incubation period ≤14 days, according to Poisson regression model (Table 4). On the other hand, higher transmissibility was found for elderly patients aged > 60 years as compared with those aged 45 years or less, for patients from Northern China than those from Central China or Southern China, and for patients who were sick before Level I response measures administration (all *P* < 0.001). There were insufficient statistical evidence of an effect on transmissibility in terms of sex, type of residence, disease severity, or underlying disease. In contrast, the analysis on the imported cases from Hubei revealed higher transmissibility among patients of the male gender, with preexisting cardiovascular and cerebrovascular diseases or other diseases (Supplementary Table 6).

**Table 4:** Factors associated with the transmissibility of COVID-19 among 525 primary cases using Poisson regression model

Factors	N	Univariate analysis		Multivariate analysis^**#**^	
		EXP(β) (95%CI)	***P***-value	EXP(β) (95%CI)	***P***-value
**Incubation**					
Normal	475	1	–	1	–
Prolonged	50	0.515 (0.331, 0.803)	0.003	0.504 (0.323, 0.786)	0.002
**Sex**					
Female	252	1	–		
Male	273	1.064 (0.869, 1.304)	0.546		
**Age, year**					
0–45	244	1	–	1	–
46–60	165	1.270 (1.002, 1.610)	0.048	1.241 (0.978, 1.575)	0.076
> 60	116	1.701 (1.320, 2.192)	< 0.001	1.826 (1.415, 2.357)	< 0.001
**Geographical Location**					
Northern	139	1	–	1	–
Central	246	0.468 (0.375, 0.583)	< 0.001	0.469 (0.377, 0.583)	< 0.001
Southern	140	0.458 (0.353, 0.594)	< 0.001	0.478 (0.368, 0.620)	< 0.001
**Type of residence**					
Urban area	354	1	–	No significant and excluded	
Rural area	171	1.375 (1.126, 1.678)	0.002		
**Underlying disease**					
None	371	1	–	No significant and excluded	
Cardia-cerebrovascular	76	1.235 (0.947, 1.610)	0.119		
Diabetes	27	1.622 (1.114, 2.363)	0.012		
Other disease	83	1.237 (0.963, 1.589)	0.096		
**Clinical severity**					
Non-severe	444	1	–	No significant and excluded	
Severe	81	1.821 (1.437, 2.309)	< 0.001		
**Epidemic phase**					
Before Level I response	76	1	–	1	–
After Level I response	449	0.436 (0.351, 0.542)	< 0.001	0.496 (0.398, 0.619)	< 0.001

^#^Factors with *P*-value greater than 0.1 in univariate analysis were excluded from the multivariate analysis

## Discussion

Based on the largest individual-level dataset with detailed epidemiological information, we determined over 10% of COVID-19 patients had a prolonged incubation period of > 14 days. The longer incubation periods seen in our patients might occur for a variety of reasons, but with older age and less severe disease more frequently seen, no matter local cases or imported cases were estimated. For the first time as we know, we found an increasing proportion of patients with prolonged incubation period along with the epidemic, which increased from 4.0 to 10.8% after Level I response measures were administered in local cases. This was accompanied by an increased median incubation period from 3.52 (95% CI: 3.11–3.98) to 6.83 (95% CI: 6.59–7.07). The results indicated that the medical observation period of 14 days that was adopted by most countries, recommended by WHO [20] was insufficient. Isolation and medical quarantine policies of 2 weeks currently in place may miss the patients with longer incubation period, for whom extra effective management should be adopted. In China, a “14 + 7 + 7” quarantine strategy had been employed for those returning from abroad since late December 2020. This includes 14-day intensive isolation and medical quarantine at the port of entry plus a 7-day medical observation at home and another 7-day health surveillance in the community, or locally stricter medical quarantine measure. Still, it is also important to weigh the potential health benefits of reducing transmission and thus case numbers against high economic and social costs that differ among countries.

Compared to those with an incubation period within 14 days, COVID-19 patients with a prolonged incubation period exceeding 14 days were significantly less severe and accompanied by shorter duration from symptom onset to discharge. This finding was in agreement with the notion that a shorter incubation period of SARS-CoV could be related to a more severe condition due to more aggressive and damaging inflammatory responses [21–24]. Consistent with previous researches [25, 26], sex did not effect on the length of incubation period. It’s otherwise notable that the elderly > 60 years old tended to have a longer incubation period than younger age groups. Aging can lead to compromised immune response including the immune response to respiratory viruses, which is often related to a longer incubation period [27, 28], a similar finding for SARS-CoV-1 in 2003 [29].

A higher proportion of patients with prolonged incubation period was observed in Southern and Central China than other parts of the country, which might be influenced by meteorological factors, e.g., temperature [30–32]. The function of the human immune system could be weakened in a comparatively colder environment such as in Northern China, which may raise the risk of being infected and lead to a short incubation period [31]. This was also supported by laboratory findings indicating that lower environment temperature might decrease the infection capacity and viral loads of SARS-COV-2 [32].

The lower transmissibility for those patients with longer incubation periods was also proposed to be related to lower viral loads, that might be decreased during intergenerational transmission.

The incubation period of COVID-19 also depended on how the disease has been acquired. The cases with a longer incubation period were more likely to be infected through the contact in public and working places compared to those with a household contact. Understandably that public and working places have wider or open space, which was related to a lower exposure intensity than household contact which more likely occured under closed settings.

In this study, we analyzed imported cases and locally exposed cases separately, because the self-reported delay from exposure to symptom onset was longer among cases imported from Hubei Province than locally exposed cases. First, the difference could result from inherent heterogeneity in exposure and immunity between travelers and residents due to the possibility that travelers were usually younger than the residents in general. In addition, recall bias could also differ, as travelers likely had more frequent close contacts in a variety of settings.

The study had limitations. Firstly, the classification of a case as a primary or secondary case based only on the time of symptom onset might lead to misclassification, since the date of symptom onset for some primary cases might be late than that of secondary cases. Secondly, we only included the confirmed cases with apparent illness, while asymptomatic cases were excluded due to their undefinable disease onset date, and inaccessible viral shedding data. Thus, our study conclusion cannot be extrapolated to asymptomatic cases. It is the first study that focused on the prolonged incubation of COVID-19 disease, disclosing the wide variation of incubation period of SARS-CoV-2 infection, which could be explained by the difference in the biological heterogeneity of the population and the control measures of certain regions or periods.

## Conclusions

The study provides evidence supporting a prolonged incubation period that exceeded 2 weeks in over 10% for COVID-19. Longer monitoring periods than 14 days for quarantine or persons potentially exposed to SARS-CoV-2 should be justified in extreme cases, especially for those elderly. This study may contribute to the COVID-19 control effort by providing an informed estimate of the incubation period, a key variable needed for informed decision-making throughout the pandemic.

## Supplementary Information

**Additional file 1: Supplementary Table 1.** Variance inflation factor (VIF) of each model. **Supplementary Table 2.** Comparison of demographic, clinical and epidemiological characteristics between COVID-19 imported cases from Hubei with an incubation period of ≤14 and > 14 days. **Supplementary Table 3.** Factors associated with incubation period among 2341 imported cases from Hubei using accelerated failure time model. **Supplementary Table 4.** Factors affecting the severity of COVID-19 among 2337 imported cases from Hubei using Logistic regression model. **Supplementary Table 5.** Multivariate logistic regression model for local cases and imported cases after excluding outliers. **Supplementary Table 6.** Factors associated with the transmissibility of COVID-19 among 1035 primary imported cases from Hubei using Poisson regression model. **Supplementary Figure 1.** The temporal pattern of reporting date of individual COVID-19 cases in the aggregated database, compared with national surveillance data outside Hubei province in China. **Supplementary Figure 2.** Estimated distributions of the incubation period COVID-19 local cases based on Weibull parametric model. **Supplementary Figure 3.** Estimated distributions of the incubation period COVID-19 local cases based on log-normal parametric model. **Supplementary Figure 4.** Estimated distributions of the incubation period COVID-19 local cases based on log-logistic parametric model. **Supplementary Figure 5.** Estimated distributions of the incubation period COVID-19 imported cases from Hubei based on Gamma parametric model. **Supplementary Figure 6.** Estimated distributions of the incubation period COVID-19 imported cases from Hubei based on Weibull parametric model. **Supplementary Figure 7.** Estimated distributions of the incubation period COVID-19 imported cases from Hubei based on log-normal parametric model. **Supplementary Figure 8.** Estimated distributions of the incubation period COVID-19 imported cases from Hubei based on log-logistic parametric model. **Supplementary Figure 9.** The incubation period in different populations profiled along epidemic period among imported cases from Hubei.

## Data Availability

Dataset of individual COVID-19 cases and the source transmission clusters are available from the open-access websites, mainly from the websites of provincial and municipal health commissions in China. Dataset of individual data on occupation, underlying diseases, and clinical severity are not publicly available but are available from the corresponding author on reasonable request.
