# Supplementary material for: Epidemiological features of COVID-19 patients with prolonged incubation period and its implications for controlling the epidemics in China
Source: BMC Public Health. 2021 Dec 9;21:2239. doi: 10.1186/s12889-021-12337-9 (PMC8655494; doi:10.1186/s12889-021-12337-9)

**Supplementary Appendix:**

**Zhi-Jie Zhang, et al. Epidemiological features of COVID-19 patients with prolonged incubation period and its implications for controlling the epidemics in China**

**Supplementary Table 1. Variance inflation factor (VIF) of each model.**

| **Factors** | Accelerated failure time model | |  | Logistic model | |  | Possion model | |
| --- | --- | --- | --- | --- | --- | --- | --- | --- |
|  | Local | Import |  | Local | Import |  | Local | Import |
| **Incubation** | - | - |  | 1.009 | 1.007 |  | 1.034 | 1.038 |
| **Sex** | 1.008 | 1.016 |  | 1.013 | 1.025 |  | 1.062 | 1.044 |
| **Age, year** | 1.052 | 1.059 |  | 1.111 | 1.116 |  | 1.213 | 1.245 |
| **Occupation** | 1.049 | 1.058 |  | 1.065 | 1.060 |  | 1.140 | 1.146 |
| **Geographical Location** | 1.013 | 1.033 |  | 1.008 | 1.031 |  | 1.027 | 1.113 |
| **Type of residence** | 1.038 | 1.024 |  | 1.101 | 1.072 |  | 1.128 | 1.065 |
| **Cardia-cerebrovascular disease** | 1.113 | 1.091 |  | 1.117 | 1.096 |  | 1.189 | 1.176 |
| **Diabetes** | 1.050 | 1.043 |  | 1.063 | 1.051 |  | 1.095 | 1.097 |
| **Other disease** | 1.019 | 1.013 |  | 1.022 | 1.016 |  | 1.053 | 1.023 |
| **Clinical severity** | - | - |  | - | - |  | 1.088 | 1.094 |
| **Epidemic phases** | 1.004 | 1.014 |  | - | - |  | 1.031 | 1.090 |
| **Infection route** | 1.008 | - |  | - | - |  | - | - |

* “-” indicates that this variable is not included in the model

**Supplementary Table 2. Comparison of demographic, clinical and epidemiological characteristics** **between COVID-19 imported cases from Hubei with an incubation period of** ≤**14 and >14 days.**

| **Characteristics** | **Number of cases (%)** | | **P-value^#^** |
| --- | --- | --- | --- |
|  | ≤**14 days** | **>14 days** |  |
| **Sex** |  |  | 0.556 |
| Male | 1371 (56.40%) | 276 (57.98%) |  |
| Female | 1060 (43.60%) | 200 (42.02%) |  |
| **Age, year** |  |  | 0.044 |
| 0–45 | 1449 (59.61%) | 263 (55.25%) |  |
| 46–60 | 677 (27.85%) | 134 (28.15%) |  |
| >60 | 305 (12.55%) | 79 (16.60%) |  |
| **Occupation**† |  |  | 0.046 |
| Manual worker* | 210 (10.80%) | 33 (8.33%) |  |
| Farmer | 228 (11.72%) | 36 (9.09%) |  |
| Office worker | 383 (19.69%) | 65 (16.41%) |  |
| Service worker | 538 (27.66%) | 124 (31.31%) |  |
| Unemployed | 586 (30.13%) | 138 (34.85%) |  |
| **Geographical Location** |  |  | 0.157 |
| Northern | 373 (15.34%) | 68 (14.29%) |  |
| Central | 828 (34.06%) | 184 (38.66%) |  |
| Southern | 1230 (50.60%) | 224 (47.06%) |  |
| **Type of residence** |  |  | 0.030 |
| Urban area | 1426 (58.66%) | 253 (53.15%) |  |
| Rural area | 1005 (41.34%) | 223 (46.85%) |  |
| **Case type** |  |  | <0.001 |
| Primary cases | 2156 (88.69%) | 348 (73.11%) |  |
| Secondary cases | 275 (11.31%) | 128 (26.89%) |  |
| **Clinical severity**† |  |  | 0.002 |
| Non-severe | 1777 (90.07%) | 381 (95.01%) |  |
| Severe | 196 (9.93%) | 20 (4.99%) |  |
| **Disease outcome** † |  |  | 1.000 |
| Discharge | 1967 (99.44%) | 400 (99.50%) |  |
| Death | 11 (0.56%) | 2 (0.50%) |  |
| **Duration from onset to discharge**^†^, median days (IQR) | 22.00 (17.00, 28.00) | 19.00 (15.00, 24.00) | <0.001 |
| **Epidemic phase** |  |  | <0.001 |
| Before Level I response | 722 (29.70%) | 30 (6.30%) |  |
| After Level I response | 1709 (70.30%) | 446 (93.70%) |  |
| **Underlying medical condition** † |  |  | 0.794 |
| None | 1553 (78.51%) | 319 (79.35%) |  |
| Cardia-cerebrovascular disease | 193 (9.76%) | 38 (9.45%) |  |
| Diabetes | 80 (4.04%) | 12 (2.99%) |  |
| Other disease | 235 (11.88%) | 48 (11.94%) |  |

# *P*-values were calculated by Pearson’s Chi-square test or Fisher's exact test, except that duration from onset to discharge were calculated by Wilcoxon sum-rank test.

* Manual worker contains construction worker, factory worker, cleaner, etc.

† Represents that the variable contains missing values.

**Supplementary Table 3. Factors associated with incubation period among 2 341 imported cases from Hubei using accelerated failure time model.**

| **Factors** | **N** | **Univariate analysis** | |  | **Multivariate analysis^#^** | |
| --- | --- | --- | --- | --- | --- | --- |
|  |  | **EXP(β) (95%CI)** | ***P*-value** |  | **EXP(β) (95%CI)** | ***P*-value** |
| **Sex** |  |  |  |  |  |  |
| Female | 1003 | 1 | - |  |  |  |
| Male | 1338 | 0.964 (0.881, 1.055) | 0.424 |  |  |  |
| **Age, year** |  |  |  |  |  |  |
| >60 | 668 | 1 | - |  | 1 | - |
| 0‒45 | 273 | 0.805 (0.694, 0.933) | 0.004 |  | 0.799 (0.692, 0.922) | 0.002 |
| 46‒60 | 1400 | 0.881 (0.750, 1.035) | 0.123 |  | 0.887 (0.762, 1.034) | 0.125 |
| **Occupation** |  |  |  |  |  |  |
| Manual worker^*^ | 243 | 1 | - |  | 1 | - |
| Farmer | 264 | 1.180 (0.969, 1.438) | 0.099 |  | 1.093 (0.910, 1.313) | 0.343 |
| Office worker | 448 | 0.831 (0.710, 0.972) | 0.021 |  | 0.935 (0.808, 1.081) | 0.361 |
| Public service worker | 662 | 1.238 (1.055, 1.453) | 0.009 |  | 1.248 (1.076, 1.448) | 0.003 |
| Unemployed | 724 | 1.117 (0.960, 1.300) | 0.154 |  | 1.177 (1.017, 1.361) | 0.028 |
| **Geographical Location** |  |  |  |  |  |  |
| Northern | 400 | 1 | - |  | 1 | - |
| Central | 943 | 0.985 (0.860, 1.128) | 0.829 |  | 0.922 (0.813, 1.044) | 0.200 |
| Southern | 998 | 0.858 (0.752, 0.978) | 0.022 |  | 0.799 (0.706, 0.903) | <0.001 |
| **Type of residence** |  |  |  |  |  |  |
| Urban area | 1226 | 1 | - |  | 1 | - |
| Rural area | 1115 | 1.493 (1.354, 1.646) | <0.001 |  | 1.417 (1.290, 1.557) | <0.001 |
| **Underlying disease** |  |  |  |  |  |  |
| None | 1837 | 1 | - |  | No significant and excluded | |
| Cardio-cerebrovascular disease | 231 | 1.160 (0.979, 1.373) | 0.086 |  |  |  |
| Diabetes | 91 | 0.818 (0.647, 1.035) | 0.094 |  |  |  |
| Other diseases | 280 | 0.993 (0.868, 1.136) | 0.915 |  |  |  |
| **Epidemic phase** |  |  |  |  |  |  |
| Before Level I response | 629 | 1 | - |  | 1 | - |
| After Level I response | 1712 | 1.736 (1.583, 1.904) | <0.001 |  | 1.838 (1.673, 2.019) | <0.001 |

# Factors with *P*-value greater than 0.1 in univariate analysis were excluded from the multivariate analysis.

* Manual worker contains construction worker, factory worker, cleaner, etc.

**Supplementary Table 4. Factors affecting the severity of COVID-19 among 2 337 imported cases from Hubei using Logistic regression model.**

| **Factors** | **N** | **Univariate analysis** | |  | **Multivariate analysis^#^** | |
| --- | --- | --- | --- | --- | --- | --- |
|  |  | **OR (95%CI)** | ***P*-value** |  | **OR (95%CI)** | ***P*-value** |
| **Incubation** |  |  |  |  |  |  |
| Normal | 1942 | 1 | - |  | 1 | - |
| Prolonged | 395 | 0.481 (0.291, 0.753) | 0.002 |  | 0.443 (0.264, 0.708) | 0.001 |
| **Sex** |  |  |  |  |  |  |
| Female | 999 | 1 | - |  |  |  |
| Male | 1338 | 1.251 (0.939, 1.676) | 0.129 |  |  |  |
| **Age, year** |  |  |  |  |  |  |
| 0–45 | 1399 | 1 | - |  | 1 | - |
| 46–60 | 667 | 3.053 (2.199, 4.257) | <0.001 |  | 2.472 (1.746, 3.508) | <0.001 |
| >60 | 271 | 5.098 (3.472, 7.464) | <0.001 |  | 3.677 (2.380, 5.644) | <0.001 |
| **Occupation** |  |  |  |  |  |  |
| Manual worker^*^ | 243 | 1 | - |  |  | |
| Farmer | 264 | 1.458 (0.821, 2.638) | 0.203 |  |  |  |
| Office worker | 448 | 0.841 (0.478, 1.508) | 0.551 |  |  |  |
| Public service worker | 661 | 0.903 (0.539, 1.563) | 0.705 |  |  |  |
| Unemployed | 721 | 1.246 (0.764, 2.115) | 0.396 |  |  |  |
| **Geographical Location** |  |  |  |  |  |  |
| Northern | 400 | 1 | - |  | 1 | - |
| Central | 942 | 0.833 (0.575, 1.220) | 0.339 |  | 0.764 (0.517, 1.141) | 0.182 |
| Southern | 995 | 0.636 (0.434, 0.942) | 0.022 |  | 0.544 (0.363, 0.822) | 0.003 |
| **Type of residence** |  |  |  |  |  |  |
| Urban area | 1224 | 1 | - |  |  |  |
| Rural area | 1113 | 1.181 (0.891, 1.566) | 0.246 |  |  |  |
| **Underlying disease** |  |  |  |  |  |  |
| None | 1833 | 1 | - |  | 1 | - |
| Cardia-cerebrovascular disease | 231 | 3.042 (2.095, 4.358) | <0.001 |  | 1.987 (1.338, 2.918) | 0.001 |
| Diabetes | 91 | 4.429 (2.701, 7.152) | <0.001 |  | 3.378 (2.051, 5.489) | <0.001 |
| Other disease | 280 | 1.295 (0.855, 1.913) | 0.207 |  | 1.155 (0.759, 1.717) | 0.488 |

# Factors with *P*-value greater than 0.1 in univariate analysis were excluded in the multivariate analysis.

* Manual worker contains construction worker, factory worker, cleaner, etc.

**Supplementary Table 5. Multivariate logistic regression model for local cases and imported cases after excluding outliers.** The attribute value frequency (AVF) was used to filter out outliers.

| **Factors** | **Local cases**  **(remove 19 outliers)** | |  | **Imported cases**  **(remove 23 outliers)** | |  |
| --- | --- | --- | --- | --- | --- | --- |
|  | **OR (95%CI)** | ***P*-value** |  | **OR (95%CI)** | ***P*-value** |  |
| **Incubation** |  |  |  |  |  |  |
| Normal | 1 | - |  | 1 | - |  |
| Prolonged | 0.374 (0.187, 0.679) | 0.003 |  | 0.400 (0.222, 0.669) | 0.001 |  |
| **Age, year** |  |  |  |  |  |  |
| 0–45 | 1 | - |  | 1 | - |  |
| 46–60 | 1.353 (0.907, 2.018) | 0.137 |  | 2.477 (1.744, 3.525) | <0.001 |  |
| >60 | 4.402 (3.015, 6.477) | <0.001 |  | 3.821 (2.458, 5.895) | <0.001 |  |
| **Geographical Location** |  |  |  |  |  |  |
| Northern | 1 | - |  | 1 | - |  |
| Central | 0.644 (0.463, 0.897) | 0.009 |  | 0.746 (0.503, 1.120) | 0.151 |  |
| Southern | 0.599 (0.399, 0.891) | 0.012 |  | 0.516 (0.341, 0.786) | 0.002 |  |
| **Underlying disease** |  |  |  |  |  |  |
| None | 1 | - |  | 1 | - |  |
| Cardia-cerebrovascular disease | 1.549 (1.061, 2.241) | 0.022 |  | 1.788 (1.180, 2.671) | 0.005 |  |
| Diabetes | 2.646 (1.555, 4.417) | <0.001 |  | 3.306 (1.905, 5.616) | <0.001 |  |
| Other disease | 1.680 (1.154, 2.414) | 0.006 |  | 0.967 (0.604, 1.492) | 0.882 |  |

**Supplementary Table 6. Factors associated with the transmissibility of COVID-19 among 1035 primary imported cases from Hubei using Poisson regression model.**

| **Factors** | **N** | **Univariate analysis** | |  | **Multivariate analysis^#^** | |
| --- | --- | --- | --- | --- | --- | --- |
|  |  | **EXP(β) (95%CI)** | ***P*-value** |  | **EXP(β) (95%CI)** | ***P*-value** |
| **Incubation** |  |  |  |  |  |  |
| Normal | 912 | 1 | - |  | 1 | - |
| Prolonged | 123 | 0.847 (0.634, 1.132) | 0.263 |  | 0.631 (0.442, 0.900) | 0.011 |
| **Sex** |  |  |  |  |  |  |
| Female | 440 | 1 | - |  | 1 | - |
| Male | 595 | 1.245 (1.023, 1.514) | 0.029 |  | 1.29 (1.058, 1.571) | 0.012 |
| **Age, year** |  |  |  |  |  |  |
| 0–45 | 612 | 1 | - |  | 1 | - |
| 46–60 | 307 | 1.559 (1.269, 1.915) | <0.001 |  | 1.495 (1.209, 1.848) | <0.001 |
| >60 | 116 | 1.777 (1.369, 2.306) | <0.001 |  | 1.605 (1.21, 2.129) | 0.001 |
| **Geographical** **Location** |  |  |  |  |  |  |
| Northern | 180 | 1 | - |  |  |  |
| Central | 423 | 0.906 (0.712, 1.154) | 0.424 |  |  |  |
| Southern | 432 | 1.034 (0.811, 1.319) | 0.785 |  |  |  |
| **Type of residence** |  |  |  |  |  |  |
| Urban area | 621 | 1 | - |  | No significant and excluded | |
| Rural area | 414 | 0.838 (0.692, 1.015) | 0.071 |  |  |  |
| **Underlying disease** |  |  |  |  |  |  |
| None | 803 | 1 | - |  | 1 | - |
| Cardia-cerebrovascular | 98 | 1.813 (1.402, 2.343) | <0.001 |  | 1.453 (1.108, 1.905) | 0.007 |
| Diabetes | 38 | 0.818 (0.503, 1.331) | 0.419 |  | 0.694 (0.427, 1.129) | 0.141 |
| Other disease | 134 | 1.479 (1.165, 1.878) | 0.001 |  | 1.444 (1.137, 1.835) | 0.003 |
| **Clinical severity** |  |  |  |  |  |  |
| Non-severe | 931 | 1 | - |  | No significant and excluded | |
| Severe | 104 | 1.531 (1.195, 1.960) | 0.001 |  |  |  |
| **Epidemic phase** |  |  |  |  |  |  |
| Before Level I response | 335 | 1 | - |  | 1 | - |
| After Level I response | 700 | 0.717 (0.596, 0.862) | <0.001 |  | 0.747 (0.619, 0.902) | 0.002 |

# Factors with *P*-value greater than 0.1 in univariate analysis were excluded from the multivariate analysis.

**Supplementary Figure 1. The temporal pattern of reporting date of individual COVID-19 cases in the aggregated database, compared with national surveillance data outside Hubei province in China.** Green bars indicated local cases with exposure date and onset date in the current database. Red bars indicated cases with exposure date and onset date in the current database. Blue bars indicated cases without exposure date and onset date in the current database. Cases outside Hubei province who were reported to the national surveillance system but not included in our analysis were represented by purple bars.


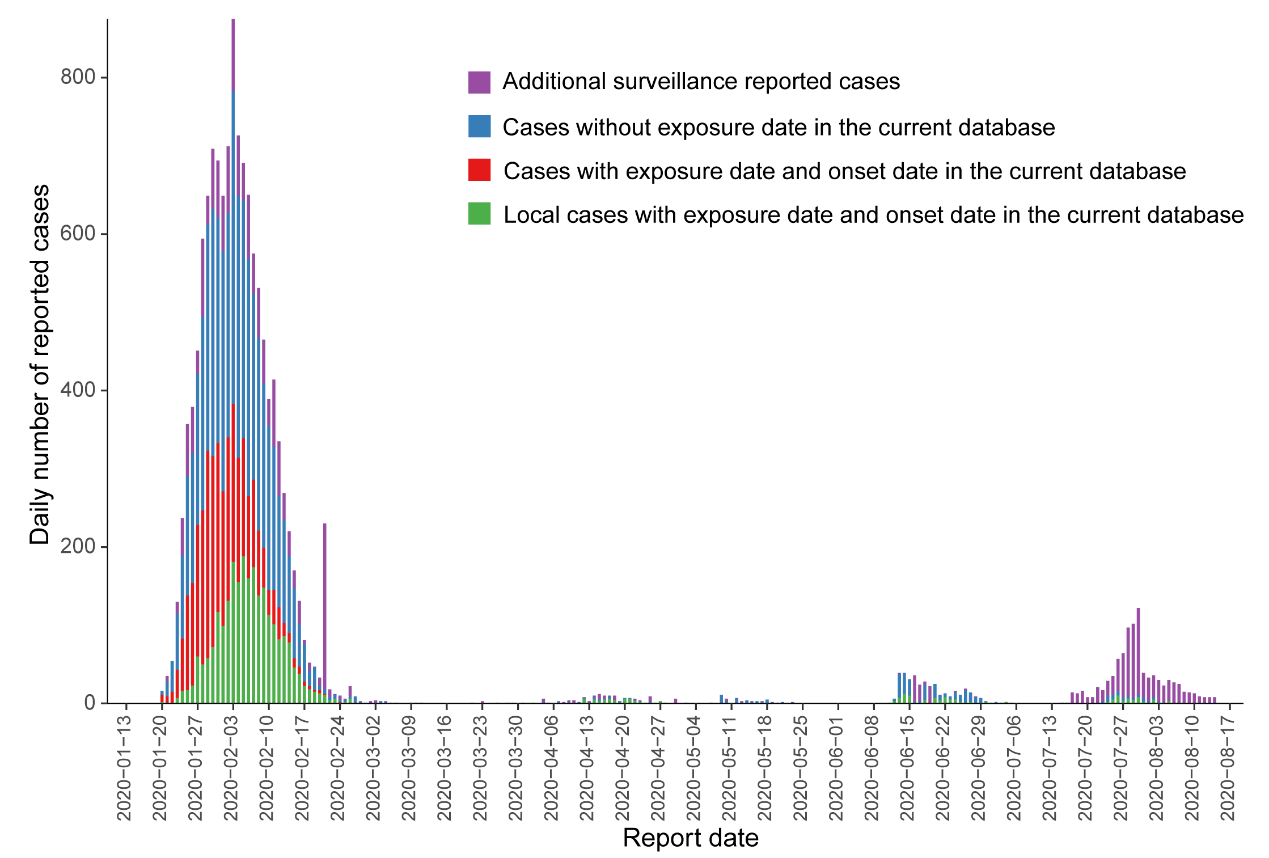


**Supplementary Figure 2. Estimated distributions of the incubation period COVID-19 local cases based on Weibull parametric model.** (A) The incubation period distribution of all cases. (B) The incubation period distribution of different age groups. (C) The incubation period distribution of different time groups from onset to discharge. (D) The incubation period distribution of different infection routes. Vertical lines indicate median of the Weibull distribution.

**
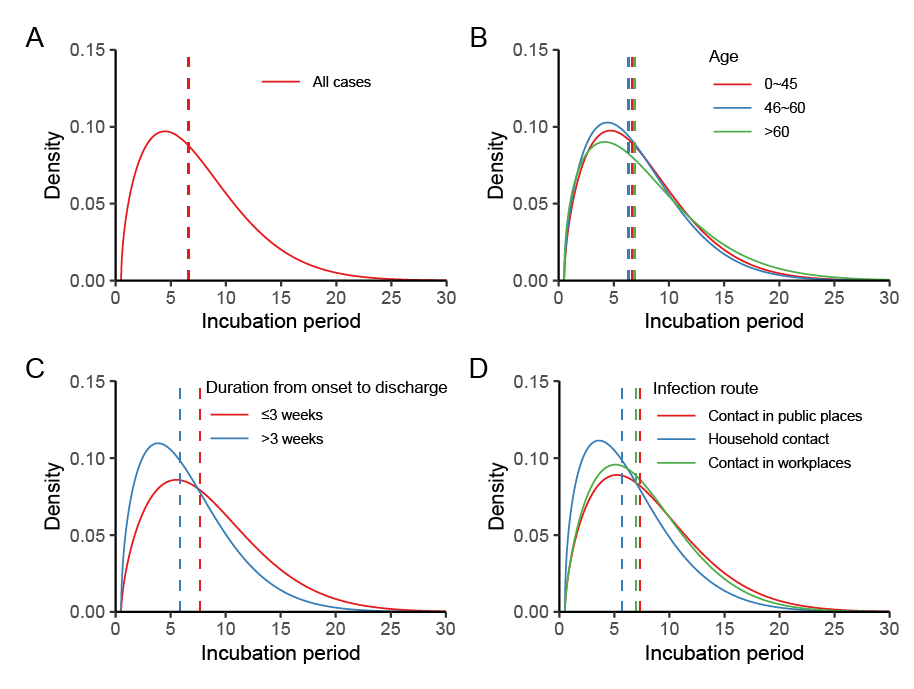
**

**Supplementary Figure 3. Estimated distributions of the incubation period COVID-19 local cases based on log-normal parametric model.** (A) The incubation period distribution of all cases. (B) The incubation period distribution of different age groups. (C) The incubation period distribution of different time groups from onset to discharge. (D) The incubation period distribution of different infection routes. Vertical lines indicate median of the log-normal distribution.

**
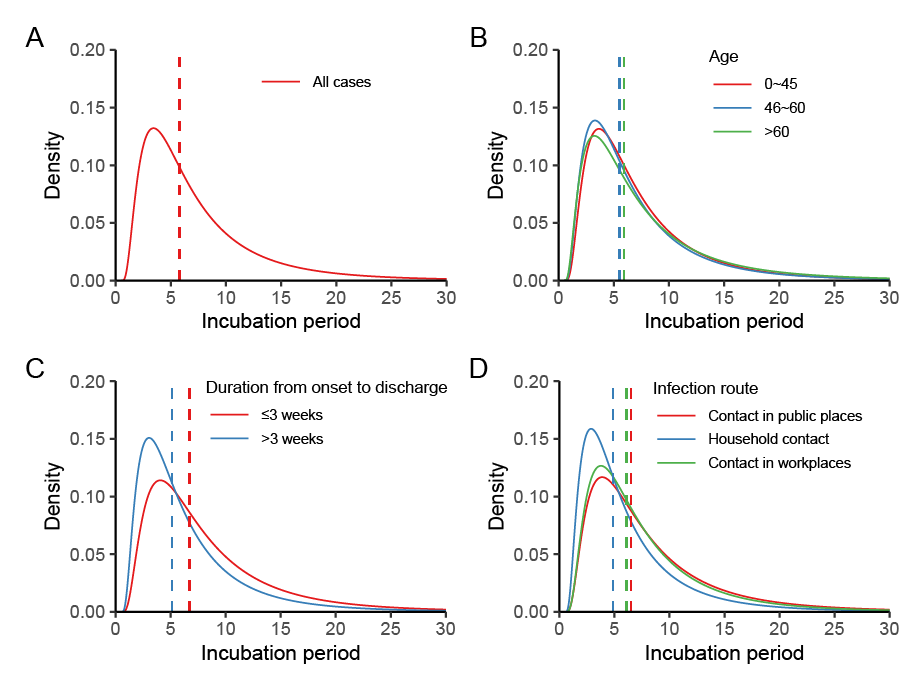
**

**Supplementary Figure 4. Estimated distributions of the incubation period COVID-19 local cases based on log-logistic parametric model.** (A) The incubation period distribution of all cases. (B) The incubation period distribution of different age groups. (C) The incubation period distribution of different time groups from onset to discharge. (D) The incubation period distribution of different infection routes. Vertical lines indicate median of the log-logistic distribution.

**
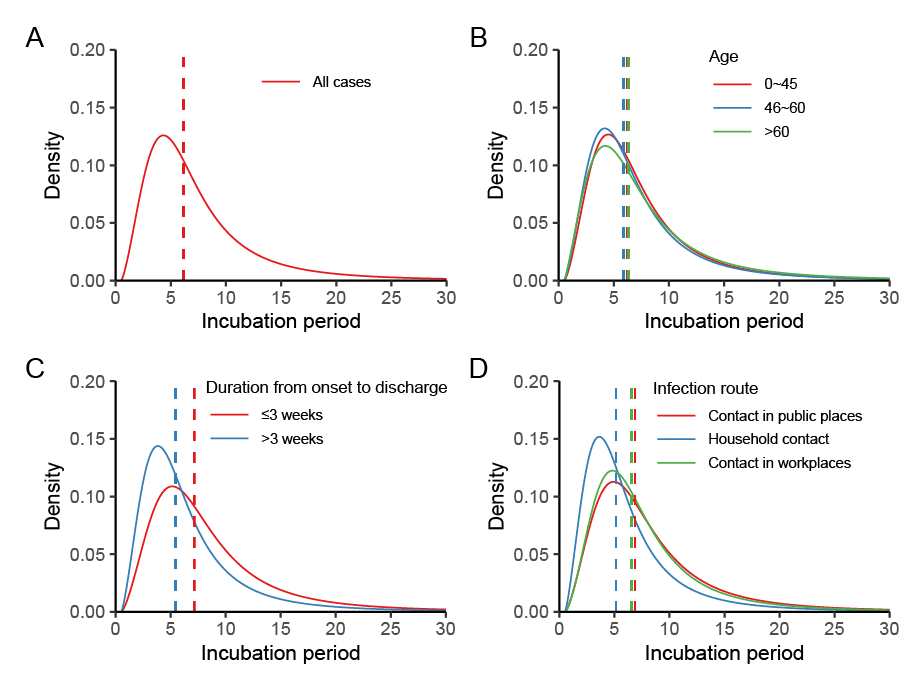
**

**Supplementary Figure 5. Estimated distributions of the incubation period COVID-19 imported cases from Hubei based on Gamma parametric model.** (A) The incubation period distribution of all cases. (B) The incubation period distribution of different age groups. (C) The incubation period distribution of different time groups from onset to discharge. Vertical lines indicate median of the Gamma distribution.


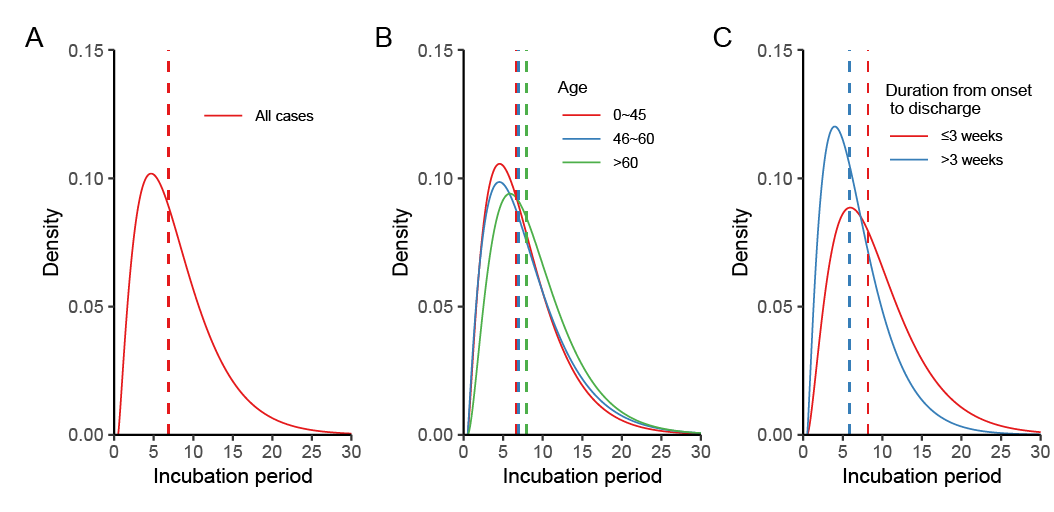


**Supplementary Figure 6. Estimated distributions of the incubation period COVID-19 imported cases from Hubei based on Weibull parametric model.** (A) The incubation period distribution of all cases. (B) The incubation period distribution of different age groups. (C) The incubation period distribution of different time groups from onset to discharge. Vertical lines indicate median of the Weibull distribution.

**
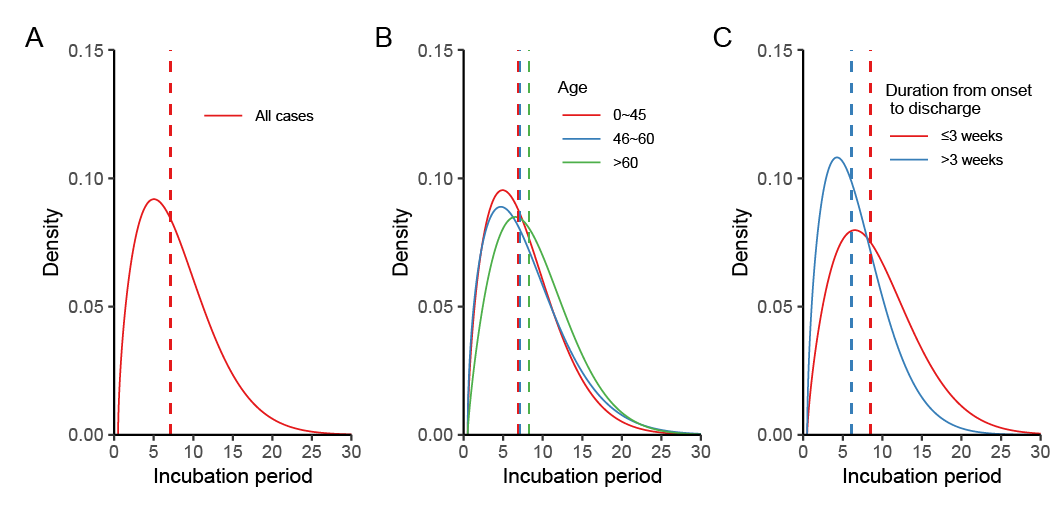
**

**Supplementary Figure 7. Estimated distributions of the incubation period COVID-19 imported cases from Hubei based on log-normal parametric model.** (A) The incubation period distribution of all cases. (B) The incubation period distribution of different age groups. (C) The incubation period distribution of different time groups from onset to discharge. Vertical lines indicate median of the log-normal distribution.

**
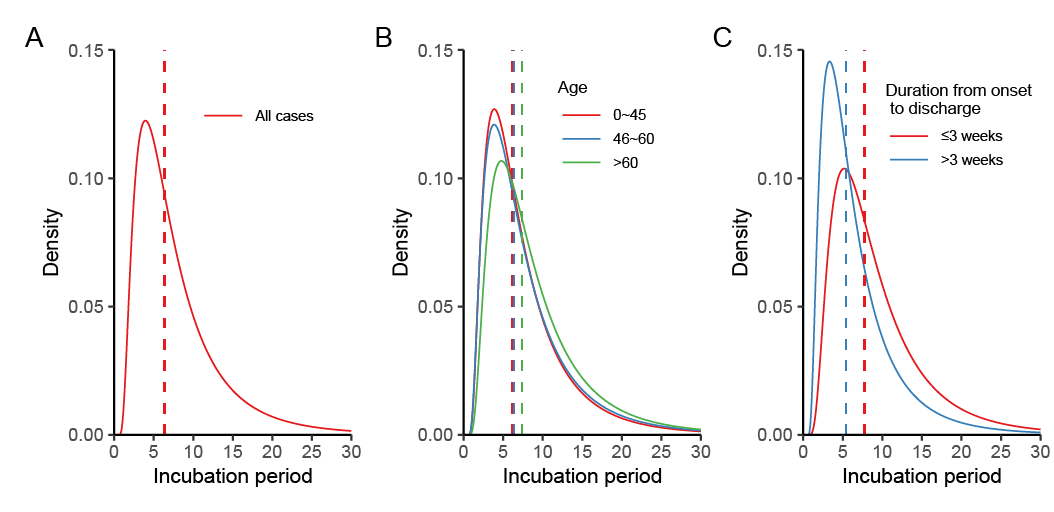
**

**Supplementary Figure 8. Estimated distributions of the incubation period COVID-19 imported cases from Hubei based on log-logistic parametric model.** (A) The incubation period distribution of all cases. (B) The incubation period distribution of different age groups. (C) The incubation period distribution of different time groups from onset to discharge. Vertical lines indicate median of the log-logistic distribution.

**
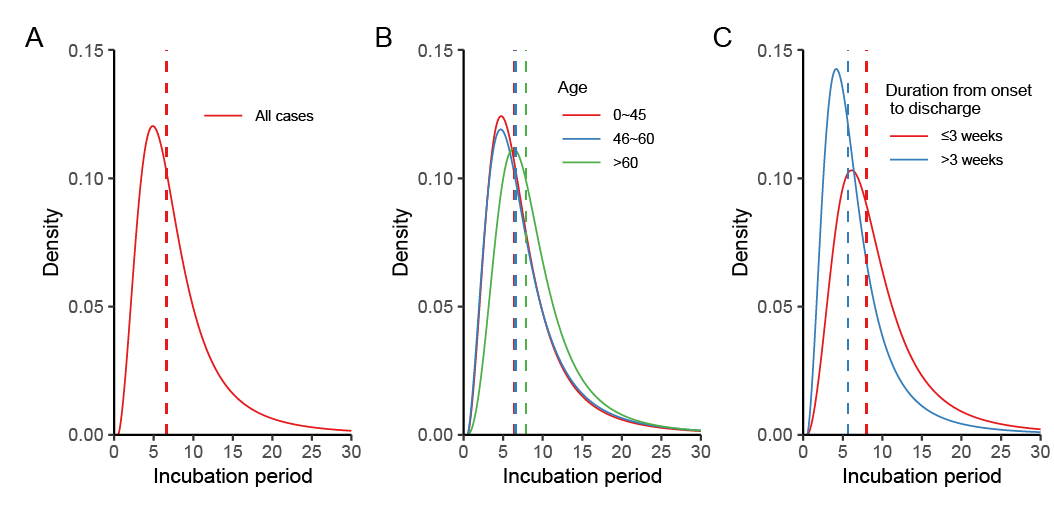
**

**Supplementary Figure 9. The incubation period in different populations profiled along epidemic period among imported cases from Hubei.** The proportion of subgroups of patients corresponded to the left axis. The incubation period estimated from subgroups of patients corresponded to the right axis.


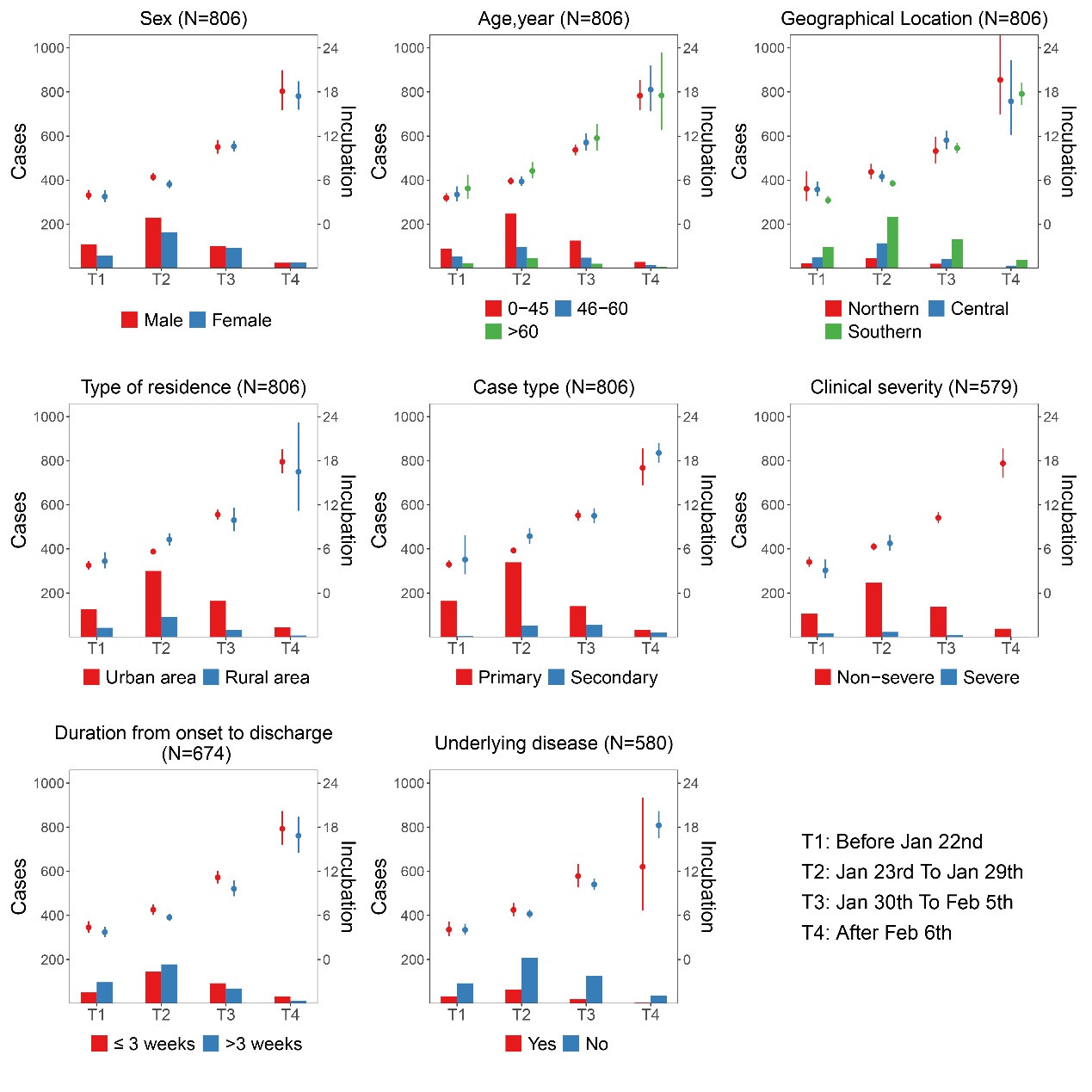

Supplement: Supplementary file 1 — Additional file 1: Supplementary Table 1. Variance inflation factor (VIF) of each model. Supplementary Table 2. Comparison of demographic, clinical and epidemiological characteristics between COVID-19 imported cases from Hubei with an incubation period of ≤14 and > 14 days. Supplementary Table 3. Factors associated with incubation period among 2341 imported cases from Hubei using accelerated failure time model. Supplementary Table 4. Factors affecting the severity of COVID-19 among 2337 imported cases from Hubei using Logistic regression model. Supplementary Table 5. Multivariate logistic regression model for local cases and imported cases after excluding outliers. Supplementary Table 6. Factors associated with the transmissibility of COVID-19 among 1035 primary imported cases from Hubei using Poisson regression model. Supplementary Figure 1. The temporal pattern of reporting date of individual COVID-19 cases in the aggregated database, compared with national surveillance data outside Hubei province in China. Supplementary Figure 2. Estimated distributions of the incubation period COVID-19 local cases based on Weibull parametric model. Supplementary Figure 3. Estimated distributions of the incubation period COVID-19 local cases based on log-normal parametric model. Supplementary Figure 4. Estimated distributions of the incubation period COVID-19 local cases based on log-logistic parametric model. Supplementary Figure 5. Estimated distributions of the incubation period COVID-19 imported cases from Hubei based on Gamma parametric model. Supplementary Figure 6. Estimated distributions of the incubation period COVID-19 imported cases from Hubei based on Weibull parametric model. Supplementary Figure 7. Estimated distributions of the incubation period COVID-19 imported cases from Hubei based on log-normal parametric model. Supplementary Figure 8. Estimated distributions of the incubation period COVID-19 imported cases from Hubei based on log-logistic parametric model. Su [file 12889_2021_12337_MOESM1_ESM.docx]
